# P-878. The Impact of Inappropriate Empirical Antibiotic Choices on Hemodynamics in Multidrug-Resistant Bacterial Sepsis

**DOI:** 10.1093/ofid/ofae631.1069

**Published:** 2025-01-29

**Authors:** Sarunya Pakapanich, Anupol Panichote, Atibordee Meesing

**Affiliations:** Khon Kaen University, Mueang, Khon Kaen, Thailand; Khon Kaen university, Khonkaen, Khon Kaen, Thailand; Khon Kaen university, Khonkaen, Khon Kaen, Thailand

## Abstract

**Background:**

Empirical antibiotics are crucial in managing sepsis, especially in multidrug-resistant (MDR) bacteria. This study aims to investigate the hemodynamic consequences of administering inappropriate empirical antibiotics to sepsis patients with MDR bacteremia.

Characteristics of the Patients

**Figure:**
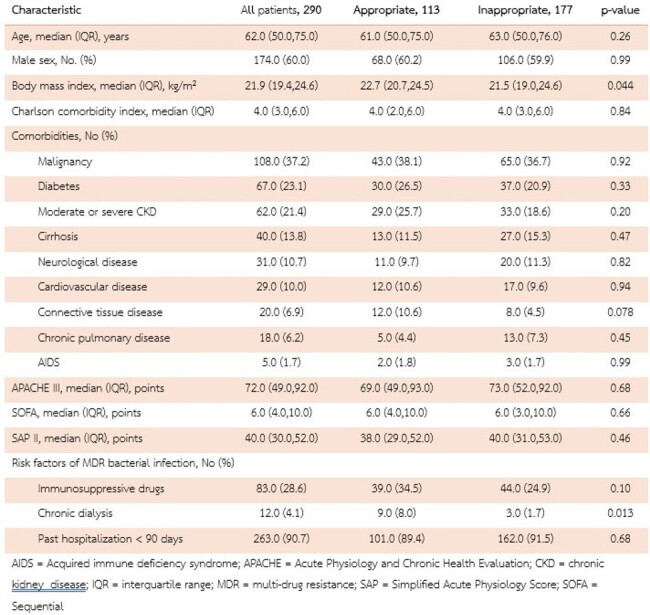

**Methods:**

This ambispective cohort study (January 1, 2018, to December 31, 2023) focused on adults over 18 with MDR septicemia.

The inappropriate empirical antibiotic was defined as patients who did not receive the first antibiotic active in vitro against the bacteria. The primary outcome was shock control rates within 3 hours after vasopressor use.

Sepsis data and shock control

**Figure:**
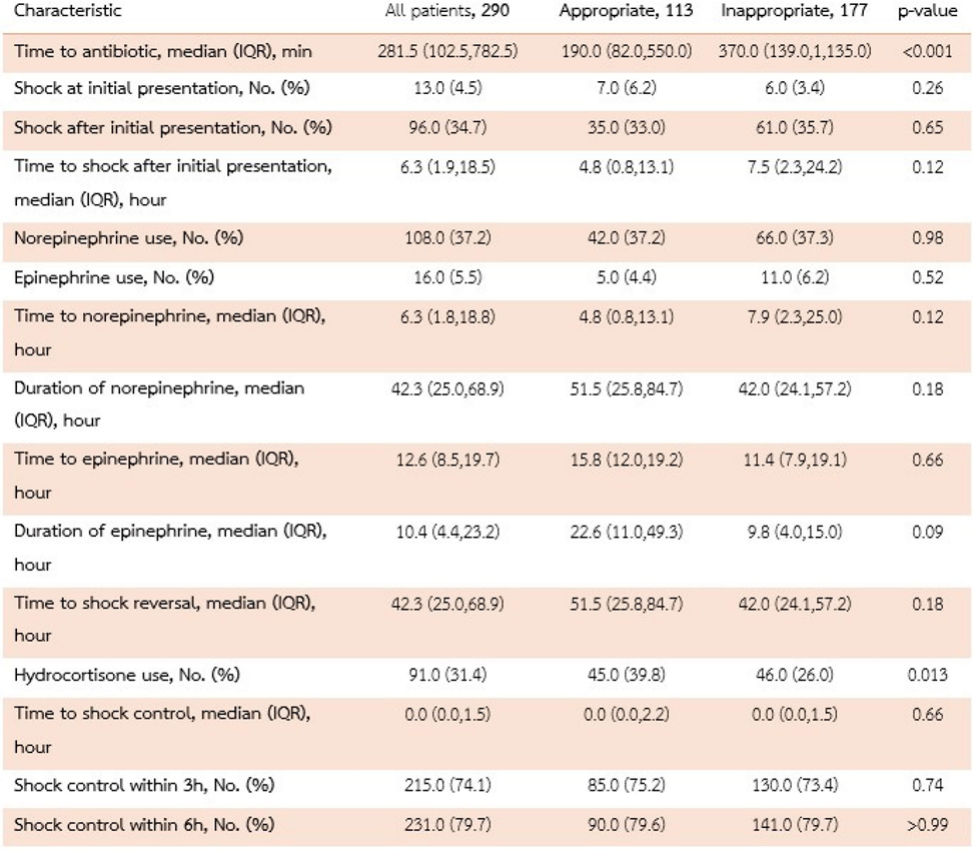

**Results:**

Our study included 290 patients; 177 (61.0%) received inappropriate empirical antibiotic therapy. *Escherichia coli* and *Klebsiella pneumoniae* were the most common pathogens. The median time to antibiotic initiation was longer in the inappropriate group (370 minutes; IQR 139–1135) than in the appropriate group (190 minutes; IQR 82–550). Shock control within 3 hours was achieved by 73.4% in the inappropriate group and 75.2% in the appropriate group, and within 6 hours by 79.7% and 79.6%, respectively. Cox regression showed no significant difference in time to shock control or 28-day mortality due to inappropriate empirical antibiotic (HR 0.82; 95%CI 0.38–1.77 for 3 hours, HR 0.87; 95%CI 0.46–1.64 for 6 hours, and HR 1.25; 95%CI 0.82–1.91 for 28-day mortality), even after adjusting for various confounders. Achieving shock control correlated with lower 28-day mortality (HR 0.17; 95%CI 0.11–0.25 for 3 hours, HR 0.15; 95%CI 0.10–0.21 for 6 hours).

Probability of shock control at 3 hours

**Figure:**
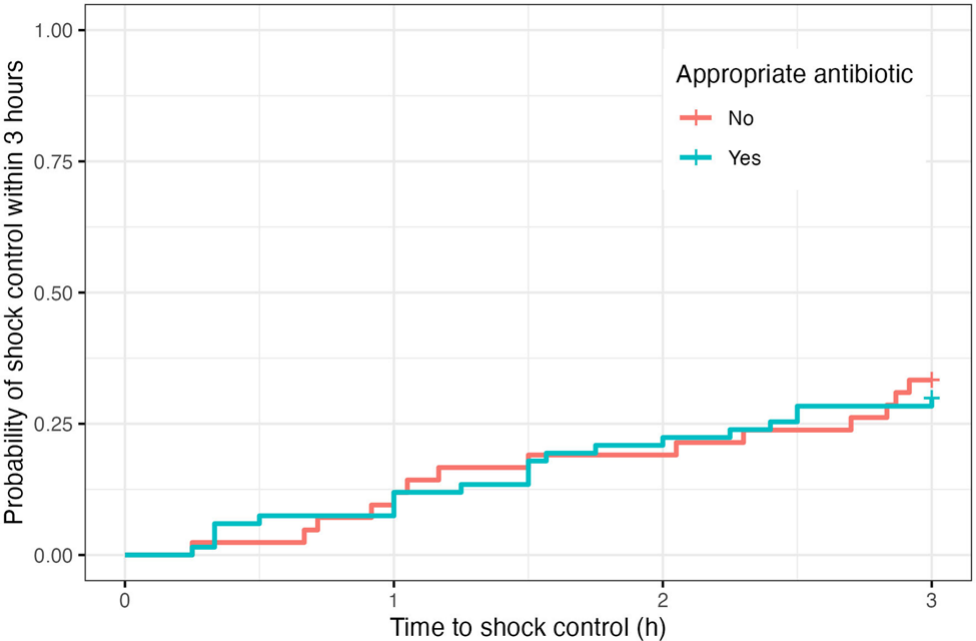

**Conclusion:**

The choice of empirical antibiotics administered was not associated with shock control or mortality outcomes.

**Disclosures:**

**All Authors**: No reported disclosures

